# Job satisfaction and social identification among paramedics in southern Poland

**DOI:** 10.3389/fpubh.2024.1422933

**Published:** 2024-06-26

**Authors:** Paweł Kukla, Maria Kózka, Patrycja Siemiginowska, Tomasz Ilczak, Magdalena Augustyn, Iwona Malinowska-Lipień

**Affiliations:** ^1^Faculty of Health Sciences, Jagiellonian University Medical College, Kraków, Poland; ^2^Institute of Applied Psychology, Faculty of Management and Social Communication, Jagiellonian University, Kraków, Poland; ^3^Department of Emergency Medicine, Faculty of Health Sciences, University of Bielsko-Biala, Bielsko-Biała, Poland

**Keywords:** job satisfaction, work engagement, social identification, psychosocial working conditions, paramedic

## Abstract

**Introduction:**

Job satisfaction, based on professional and non-professional factors and individual characteristics of employees, is an important element influencing both the quality of care provided and employee turnover.

**Material and method:**

The study included 137 paramedics employed in field teams and hospital emergency departments. The Job Satisfaction Scale (SSP), the Minnesota Job Satisfaction Questionnaire (MSQ), the Utrecht Work Engagement Scale (UWES), and the Three Dimensional Strength of Group Identification Scale (TSIG) were used to collect the data.

**Results:**

The average job satisfaction score measured with SSP in the studied group of paramedics was 24.50 and the average job satisfaction score measured with MSQ was 74.16. The average value of the group identification in the study sample was 61.15. Of the three subscales, the highest scores were obtained in the affect toward the group subscale −22.44, and the lowest in the cognitive centrality subscale −18.78. The analysis showed that job satisfaction positively correlated with social identification (*r* = 0.43) and the ingroup ties (*r* = 0.43), cognitive centrality (*r* = 0.34) and ingroup affect (*r* = 0.37).

**Conclusions:**

The studied group of paramedics showed moderate job satisfaction (measured with SSP) and work engagement, with a simultaneous high level of job satisfaction (measured with MSQ) and social identification with the professional group. Social identification of studied paramedics varied depending on gender. Women showed higher levels of cognitive centrality, which might mean that they might have had greater need to categorize themselves as paramedics.

## Introduction

In Poland, as in many other countries, the entire emergency medical system in pre-hospital care is based on paramedics, a profession that is the first link in pre-hospital care. Their work environment is associated with psychophysical threats that result in increased levels of stress, burnout, sickness absence, which also results in employee turnover and reduced job satisfaction (1). A paramedic performs medical services in unpredictable conditions. There is necessity to quickly make accurate decisions (2).

In many countries around the world, the problem of lack of job satisfaction in the professional group of paramedics is beginning to be noticed. Workload has caused a significant increase in the rate of depression and dissatisfaction in this professional group. In the USA, the annual turnover of paramedics was approximately 10% (3). In Israel, the turnover rate was even higher, reaching 42% after 2 years, and 90% with work experience of more than 10 years (4). In Germany, 54% of paramedics declared their intention to leave the profession, and 46% were dissatisfied with their work (5). The departure of experienced paramedics with many years of work experience is a threat not only to employers, but also to other employees due to the disruption of personal relationships (6). Paramedics' job is characterized by numerous stressors related to the necessity to come into contact with, among others: victims of traffic accidents in critical condition, seriously injured children and young people. In this profession there is also a lot of time pressure, which is an important psychologically burdensome factor, constant high physical effort caused by working in a two-person team, as well as work in special conditions and external threats that cause a feeling of anxiety about one's health and life while providing help.

Both internal and external stressors are important determinants of job satisfaction (7). An employee's job satisfaction is most often influenced by professional, non-professional and individual factors related to the employee. Professional factors include: salary, opportunity for professional development (promotion), opportunity for personal development, achievements, recognition and respect from superiors, independence (level of autonomy), supervision (with regard to the level of control), working conditions (e.g., equipment, working time, social benefits), team cooperation (e.g., nature of bonds with other people, level of competition), stress (resulting from the nature of work or role conflicts). Non-professional and individual factors related to the employee include: health, age gender cognitive abilities, length of service, and form of employment (8). An employee who is satisfied with his or her job is much more likely to show initiative at work, be loyal to the employer, and to have lower level of absenteeism (1). Job satisfaction and work engagement are factors that motivate employees and lead to positive attitudes toward work and high levels of individual performance (9). Paramedic's identity can be understood as a person's sense of self and relationships with others, which are influenced by professional characteristics, norms and values. According to Godfrey and Young (10), the professional identity of a paramedic was the result of his or her individual thinking, actions and professional sense (10). Social identification means identifying with another person or group, i.e., accepting the values and beliefs of other people (10). Social identification that shapes professional identity is a continuous and multi-stage process. Individuals build their professional identity in relation to perceptions of the expectations of those around them, including educators and mentors, colleagues, patients, employers and policy makers, as well as those outside their professional lives and society in general (11). According to Chomatowska et al., the professional identity of paramedics is shaped by the values and motivations which determine the choice of the field of study and the acquired competences (12). Social identification is a process that occurs simultaneously at both the individual level (psychological adaptation) and the collective level (achieving acceptance and full participation in the professional environment), and therefore, research and measurement of professional identity are not easy tasks (13). Social identity theory is important in the development of professional identity which has interdisciplinary characteristics in relation to health care system employees (14). Yazdannik et al. believed that professional identity was a form of social identity describing how a professional group creates ideals and values that were common to professionals of this group (15). According to many authors, one's own professional group can be an important factor in shaping professional identity (16, 17).

### Objective of the work

The aim of the study was to assess the relationship between job satisfaction and social identification among paramedics.

## Materials and methods

### Study design and respondents

The research was carried out from May 2020 to February 2021 after obtaining the consent of the Bioethics Committee of the Jagiellonian University (No. 1072.6120.79.2018), the consent of the heads of the units where the research was conducted and the consent of the study participants. All participants gave written informed consent in accordance with the Declaration of Helsinki. The study sample consisted of paramedics employed in the units of the State Emergency Medical System in the area covering the voivodeships located in the south of Poland, i.e., the Subcarpathia and Lesser Poland. At the first stage of sample selection, units of the State Emergency Medical Services system (medical rescue teams, hospital emergency departments) were randomly selected. The sampling frame was the Register of State Emergency Medical Services (18). At the second stage of selection, paramedics were randomly selected. The selection frame was a list of paramedics in the selected units. In the third stage, randomly selected paramedics were invited to participate in the study. After they gave their written consent, the questionnaires were issued with a return envelope so that participation in the study ensured the anonymity of the respondent. Then the completed questionnaire was put by the participant into a box located in her/his workplace. The size of the trial was calculated using the method of covariance structure modeling, and the minimum size of the trial for our study was 96. The minimum size of the group was calculated by assuming the size of the general group-−22,481; the estimated fraction size (p)−50%; the significance level (α)−5% (0.05); and the permissible error (e)−10%. A total of 370 paramedics out of the 20,481 paramedics registered in Poland agreed to voluntarily participate in the study. Of the original 370, 137 of them were qualified to undergo the analysis.

It took research participants on average ~26 min (+/−12 min) to complete the set of research tools.

### Research tools

The study used a diagnostic methods divided into three sections: the first part concerned the assessment of job satisfaction, in which standardized questionnaires assessing job satisfaction were used, i.e., the Job Satisfaction Scale (SSP), the Minnesota Job Satisfaction Questionnaire (MSQ) and the Utrecht Work Engagement Scale (UWES). Second part concerned the assessment of social identification, in which the Three Dimensional Strength of Group Identification Scale (TSIG) was used. Third part was an original survey questionnaire containing questions regarding sociodemographic data (i.e., age, gender, education level, marital status, place of residence, having children) and data regarding the professional situation (i.e., place of work, work experience, sickness absence).

Job Satisfaction Scale (SSP) by Zalewska (19) concerns the conscious cognitive evaluation of work based on personal criteria. It consists of five statements: 1/“in many respects my job is close to ideal”, 2/“I have great working conditions”, 3/“I am satisfied with my job”, 4/“so far I have managed to achieve what I wanted” and 5/“if I had to decide again, I would choose the same job” (19). Each statement is assessed using a 7-point Likert scale, i.e., from 1 strongly disagree to 7 strongly agree. The higher the total obtained score, the more satisfied the respondent is with work. The internal consistency of the scale measured by Cronbach's alpha coefficient ranges from 0.814 to 0.888, and in the tested sample it was 0.816. The theoretical minimum and maximum values of the scale are 5 and 35, respectively, and the theoretical mean is 20 (19).

The Minnesota Satisfaction Questionnaire (MSQ) developed by Weiss et al. (20) consists of 20 statements that describe various aspects of work. For each of the 20 items, the respondent selects one answer from five possible ones: “very dissatisfied”, “dissatisfied”, “neither yes nor no”, “satisfied”, “very satisfied”. Depending on the category, each answer is assigned a value from 1 to 5. After summing up all the answers, the overall score is calculated (20). The Cronbach's alpha coefficient of the original tool ranged from 0.84 to 0.91, and in the study sample it was 0.895. The higher the total score, the greater the job satisfaction. The theoretical minimum and maximum values of the MSQ are 20 and 100. respectively, the theoretical mean is 60.

The Utrecht Work Engagement Scale (UWES) developed by Schaufeli and Bakker contains 17 statements rated on a 7-point response scale from 0 (“never”) to 6 (“always/every day”). The UWES provides general score as well as scores on three subscales: vigor, dedication and absorption. Vigor is assessed by six statements relating to high energy levels, resilience while working, willingness to invest efforts, perseverance and tenacity while facing difficulties. Dedication is assessed using five statements that are related to the sense of meaning in the work performed, its purposefulness, significance and feeling enthusiasm, inspiration, pride and challenge while working, being dedicated to it. Absorption is assessed by six statements that refer to the feeling of being completely absorbed by one's work to such an extent that one forgets about the passage of time and other things around him or her, so that it is difficult to break away from the activities performed. It refers to being fully concentrated and having difficulties with detaching oneself from work as time passes quickly when the person in involved in his or her work. Cronbach's alpha coefficients of the overall score range from 0.91 to 0.96, and of the individual subscales the Cronbach's alpha were: 0.83; 0.92; 0.82, respectively. In the examined sample, the following coefficients were obtained: 0.916 for the general scale and for the subscales, respectively: 0.778; 0.828; 0.825. The higher the score, the greater the work engagement. The minimum theoretical value is 0, the maximum is 102, the theoretical mean is 51. In the subscales of vigor and absorption, the theoretical minimum and maximum scores were 0 and 36, respectively, and the theoretical mean was 18, while in the subscale of dedication, the theoretical minimum score was 0 and the maximum 30 and the theoretical average was 15.

To assess social identification, the Three-Dimensional Strength of Group Identification Scale (TSIG) by Cameron J. was used. The scale measures the overall strength of social identification, specifying values for three components of identification. It consists of three subscales: ingroup ties, cognitive centrality, and ingroup affect. Cronbach's alpha coefficients of ingroup ties scale range from 0.76 to 0.84, of the cognitive centrality scale 0.96–0.78, and of the ingroup affect scale 0.77–0.82. In the examined sample, the Cronbach's alpha coefficient of the scale was 0.732, and of the subscales: 0.686; 0.607; 0.749. The higher the score, the greater the social identification. The social identification subscales allow to describe the ingroup ties (the higher the score, the stronger the bonds), the cognitive centrality of one's own group (the higher the score, the more important the group for a given person) and the ingroup affect (the higher the score, the more positive the affect). The theoretical minimum and maximum values for the entire scale are 12 and 84, respectively, and the theoretical mean is 48. For all three subscales, the theoretical minimum scores were 4, and maximum scores were 28, and the theoretical means were 16 (21).

### Statistical analysis

The results were analyzed using the IBM SPSS 8 package. In the case of quantitative variables, descriptive statistics methods were used, such as: arithmetic mean ($\bar{x}$), standard deviation (SD), minimum (Min), maximum (Max), median (Me). Qualitative variables were presented as the number (n) and frequency (%). The analysis of the significance of differences between mean values in the compared groups was carried out while maintaining the applicable rules for the selection of statistical tests. For this purpose, the distribution of the studied quantitative variables was assessed using the Kolmogorov-Smirnov test. Student's *t*-test was used to assess the significance of differences between two groups. Linear regression analysis was also performed. In all analyses, effects for which the p probability value was lower than the assumed significance level of α = 0.05 (*p* < 0.05) were considered significant.

## Results

The research was conducted among 137 paramedics from two voivodeships: Lesser Poland (*n* = 69, 50.4%) and Subcarpathian (*n* = 68, 49.6%). The overall work experience of the respondents ranged from 0.5 years to 45 years (*x̄* = 17.08 years; SD = 11.17 years), while in the position of a paramedic it ranged from 0.5 years to 38 years (*x̄* = 11.31 years; SD = 7.42 years). They have worked in their current job for 0.5 years to 38 years (*x̄* = 11.39 years; SD = 9.45 years). Of the respondents, nearly one third held managerial positions (*n* = 43.; 31.4%). Women constituted 12.4% (*n* = 17), and men 87.6% of respondents (*n* = 120). The age of the respondents ranged from 24 to 63 years (*x̄* = 39.34 years; SD = 10.19 years). 37.9% of respondents lived in a village (*n* = 52), a small town with up to 50.000 inhabitants −28.5% (*n* = 39), the remaining 33.6% (*n* = 46) lived in medium-sized or large cities. 78.3% (*n* = 107) of the surveyed paramedics were in stable formal and informal relationships. 72.3% (*n* = 99) of the respondents declared having children, mainly two children (*n* = 56; 40.9%). The majority of the surveyed group had higher education level and a bachelor's degree in emergency medical services (*n* = 91; 66.4%). The surveyed paramedics worked in the State Emergency Medical Service system: in Hospital Emergency Departments (HED) (*n* = 12; 8.8%), emergency medical teams (EMT) (*n* = 95; 69.3%) or in both units: HED and EMS (*n* = 30; 21.9%). Paramedics worked from 7 to 144 h per week (x = 63.31, SD = 22.74), with only 9.5% of paramedics (*n* = 13) working up to 40 h a week, and 45.3% of paramedics working between 40 and 60 h a week (*n* = 62), between 60 and 80 h −24.1% (*n* = 33), and over 80 hours a week −21.2% of respondents (*n* = 29). 65.7% (*n* = 90) of paramedics declared no sickness absence during the year preceding the study. Most often, paramedics were on sick leave lasting from 1 to 10 days (*n* = 29; 21.2%) (Table 1).

**Table 1:** Characteristics of the study sample.

**Characteristics of the study sample**		***N***	**%**
Gender	Men	120	87.6
	Women	17	12.4
Age	24–34	48	35.0
	35–45	53	38.7
	46–55	26	19.0
	56–63	10	7.3
Place of residence	Village	52	37.9
	Town up to 50,000 inhabitants	39	28.5
	Medium or large city	46	33.6
Marital status	Formal (married)	99	71.5
	Informal	8	5.8
	Divorced/separated	7	5.10
	Single	22	16.1
	Widowed	1	0.72
Education level	Secondary school	46	33.6
	Bachelor's degree	91	66.4
Workplace	Hospital Emergency Department (ED)	12	8.8
	Emergency Medical Team (EMS)	95	69.3
	Hospital Emergency Department/ Emergency Medical Team	30	21.9
Number of hours worked per week	≤ 40 hours	13	9.5
	40–60 hours	62	45.3
	60–80 hours	33	24.1
	≥ 80 hours	29	21.2
Sickness absence during the previous year	0 days	90	65.7
	1–10 days	29	21.2
	≥14 days	18	13.1

N, number; %, percent.

The average result of the Job Satisfaction Scale (SSP) in the studied group of paramedics was 24.50 (SD = 5.35; Me = 25; Mi*n* = 8; Max = 35). This result indicates average job satisfaction (the cognitive aspect of it). The average result of the Minnesota Satisfaction Questionnaire (MSQ) in the group of paramedics was 74.16 (SD = 9.60; Me = 73, Mi*n* = 51, Max = 100), which indicates high job satisfaction. The overall result of the work engagement measured by the Utrecht Work Engagement Scale (UWES) in the study sample of paramedics was 66.86 (SD = 14.66; Me = 66, Mi*n* = 29, Max=102). The highest results were observed in the vigor subscale −23.93 (SD=5.20) and the lowest in the absorption subscale −21.35 (SD = 6.14). The results indicate average level of work engagement among the studied paramedics (Table 2).

**Table 2:** Descriptive statistics of the results of work engagement measured with the Utrecht Work Engagement Scale (UWES).

**Subscale**	**Mean ($\bar{x}$)**	**Standard deviation (*SD*)**	**Median (*Me*)**	**Min**	**Max**
Work engagement (overall result)	66.86	14.66	66	29	102
Vigor	23.93	5.20	24	10	36
Dedication	21.58	4.95	22	8	30
Absorption	21.35	6.14	21	6	36

The average value of the social identification (TSIG) in the study sample was 61.15 (SD = 11.01). Of the three subscales, the highest scores were obtained in the ingroup affect subscale −22.44 (SD = 4.17), and the lowest in the cognitive centrality subscale −18.78 (SD = 4.24). The results indicate high social identification in terms of the overall score and ingroup affect The results in the cognitive centrality subscale in the studied group were average, which indicates a moderate belief in the importance of the group of paramedics for the study participants. The analysis showed that the paramedics who took part in the study identified highly with their professional group, especially in the terms of ingroup affect, meaning that they had positive emotional attitude toward colleagues who were also paramedics (Table 3).

**Table 3:** Three-Dimensional Strength of Group Identification Scale (TSIG)—descriptive statistics of the overall score and subscales of the tool.

**Subscale**	**Mean ($\bar{x}$)**	**Standard deviation (*SD*)**	**Median (*Me*)**	**Min**	**Max**
Social identification (overall result)	61.15	11.01	61	25	84
Ingroup ties	20.41	6.23	20	8	28
Cognitive centrality	18.78	4.24	18	5	28
Ingroup affect	22.44	4.17	23	9	28

Min, minimum; Max, maximum.

The analysis showed a statistically significant relationship between gender and cognitive centrality, i.e., the component of professional social identification, which indicates how important being part of a group was for a given individual. A significantly higher cognitive centrality scores were found in women when compared to men (21.41 vs. 18.41; *p* = 0.003) (Table 4).

**Table 4:** Assessment of gender differences in the context of job satisfaction (measured with SSP and MSQ) work engagement (measured with UWES) and social identification (measured with TSIG).

	**Gender**	***N***	**Mean $\left(\bar{x}\right)$**	**Standard deviation (*SD*)**	***p***
**Professional satisfaction**					
Job satisfaction (SSP)	Woman	17	24.706	7.122	*p* > 0.05
	Man	120	24.467	5.084	
Job satisfaction (MSQ)	Woman	17	72.176	12.340	*p* > 0.05
	Man	120	74.441	9.172	
Work engagement	Woman	17	66.412	17.066	*p* > 0.05
	Man	120	66.924	14.370	
Vigor	Woman	17	22.941	6.230	*p* > 0.05
	Man	120	24.076	5.053	
Dedication	Woman	17	21.176	5.040	*p* > 0.05
	Man	120	21.639	4.957	
Absorption	Woman	17	22.294	7.218	*p* > 0.05
	Man	120	21.210	5.989	
**Social identification**					
Social identification	Woman	17	65.882	11.931	*p* > 0.05
	Man	120	60.899	11,595	
Ingroup ties	Woman	17	21.353	4.595	*p* > 0.05
	Man	120	20.275	6.431	
Cognitive centrality	Woman	17	21.412	4.094	*p* = 0.003
	Man	120	18.408	4.139	
Ingroup affect	Woman	17	23.118	4.622	*p* > 0.05
	Man	120	22.345	4.108	

N, number; %, percent.

There was no correlation between the age of the respondents and professional satisfaction or social identification (*p* > 0.05) (Table 5).

**Table 5:** Assessment of the relationship between job satisfaction, social identification and the age of the respondents.

	**Age**	**N**	**Mean $\left(\bar{x}\right)$**	**Standard deviation (*SD*)**	***p***
**Professional satisfaction**					
Job satisfaction (SSP)	24–34	48	23.479	5.359	*p* > 0.05
	35–45	53	25.340	5.103	
	46–63	36	24.611	5.597	
Job satisfaction (MSQ)	24–34	48	72.125	9.571	*p* > 0.05
	35–45	53	74.792	9.222	
	46–63	36	76.029	9.983	
Work engagement	24–34	48	66.438	13,710	*p* > 0.05
	35–45	53	65.264	13.820	
	46–63	36	69.857	16.983	
Vigor	24–34	48	24.208	4.519	*p* > 0.05
	35–45	53	22.792	5.336	
	46–63	36	25.286	5.623	
Dedication	24–34	48	21.417	4.802	*p* > 0.05
	35–45	53	21.094	4.974	
	46–63	36	22.543	5.124	
Absorption	24–34	48	20.813	5.927	*p* > 0.05
	35–45	53	21.377	5.167	
	46–63	36	22.029	7.694	
**Social identification**					
Social identification	24–34	48	60.426	12.072	*p* > 0.05
	35–45	53	61.472	9.756	
	46–63	36	63.028	13.857	
Ingroup ties	24–34	48	20.083	4.681	*p* > 0.05
	35–45	53	20.038	4.132	
	46–63	36	21.389	9.729	
Cognitive centrality	24–34	48	18.500	4.749	*p* > 0.05
	35–45	53	18.830	3.321	
	46–63	36	19.083	4.783	
Ingroup affect	24–34	48	22.170	4.784	*p* > 0.05
	35–45	53	22.604	3.743	
	46–63	36	22.556	3.982	

The analysis showed that job satisfaction positively correlated with social identification (*r* = 0.43) and the ingroup ties (*r* = 0.43), cognitive centrality (*r* = 0.34), and ingroup affect (*r* = 0.37). The analysis showed that social identification was positively related to job satisfaction.

Moreover, it was shown that social identification positively correlated with vigor (*r* = 0.40), dedication, work engagement (*r* = 0.36) and job satisfaction. The ingroup ties correlated with vigor (*r* = 0.34) and dedication (*r* = 0.32). Cognitive centrality positively correlated with work engagement (*r* = 0.41), vigor (*r* = 0.38), dedication (*r* = 0.41) and absorption (*r* = 0.33). The ingroup affect component of social identification correlated positively with vigor (the energetic component of work engagement) (*r* = 0.36). The analysis showed that social identification was positively associated with job satisfaction and work engagement (Table 6).

**Table 6:** Pearson's r correlations of the social identification scale and its subscales with job satisfaction, and work engagement.

**Variables**	**Social identification**	**Ingroup ties**	**Cognitive centrality**	**Ingroup affect**
Job satisfaction (MSQ)	0.30^**^	0.27^**^	0.26^**^	0.28^**^
Work engagement	0.36^**^	0.28^**^	0.41^**^	0.28^**^
Vigor	0.40^**^	0.34^*^	0.38^**^	0.36^**^
Dedication	0.39^**^	0.32^**^	0.41^**^	0.29^**^
Absorption	0.22^*^	0.13	0.33^**^	0.13

^*^p < 0.05 ^**^p < 0.01.

In order to verify the differences between social identification and job satisfaction and work engagement, the surveyed paramedics were divided according to the median (Me = 61) into two groups: paramedics with a higher and lower level of social identification. The analysis showed that the level of social identification among the surveyed paramedics differentiates almost all variables. This means that this variable might play an important role in terms of job satisfaction, and work engagement.

Paramedics who identified stronger with their professional group when compared to those who identified with it to a lesser extent had significantly higher job satisfaction scores measured by SSP, and therefore their overall cognitive assessment of job satisfaction was significantly higher than that of their colleagues who were less identified with the professional group of paramedics. Paramedics who identified stronger with their group, when compared to paramedics who identified with their group to a lesser extent, had also significantly higher job satisfaction scores (measured by MSQ) with various aspects of work. People who identified with their group to a lesser extent experienced significantly lower job satisfaction measured by MSQ. Paramedics with higher levels of social identification experienced significantly higher levels of work engagement when compared to paramedics with lower level of social identification. Employees who identified stronger with the professional group of paramedics was significantly more involved in their duties than their colleagues who identified with other paramedics less. The analysis showed that people with higher level of social identification, when compared to people with a lower level of social identification, reported higher results of job satisfaction (measured both by SPP and MSQ) and work engagement (Table 7).

**Table 7:** Results of the student's t-test in the study sample regarding the studied variables.

**Variables**	**Level of social identification**	** $\bar{x}$ **	***t statistics***	***df***	**Significance level *p***
Job satisfaction (SSP)	Lower social identification	22.63	−4.231	134	0.001
		Higher social identification	26.29			
Job satisfaction (MSQ)	Lower social identification	71.52	−3.130	132	0.002
		Higher social identification	76.53			
Work engagement	Lower social identification	61.28	−4.613	133	0.001
		Higher social identification	72.13			

To verify whether and to what extent the level of job satisfaction in people with different levels of social identification can be explained by work engagement, a regression analysis was performed in both groups of employees (with lower and higher levels of social identification). The results obtained indicate different levels of explained variance in job satisfaction results among paramedics with different levels of social identification. In paramedics with lower social identification, work engagement explained ~24.8% of the variance (variability) in job satisfaction scores (Model 1), while in paramedics with higher social identification, work engagement could explain up to 26.6% of the variability in job satisfaction scores (Model 2) (Table 8).

**Table 8:** Linear regression models of job satisfaction among paramedics with lower and higher levels of social identification.

**Level of social identification**	**Regression model**	**F**	**Significance**	**R**	**R^2^**	**Corrected R^2^**	**Predictors**	**B**	**Beta**	**t**	***p***
Lower social identification	Model 1	22.722	0.001	0.509	0.259	0.248	Constant	11.242		4.610	0.001
							Work engagement	0.185	0.509	4.767	0.001
Higher social identification	Model 2	25.322	0.001	0.527	0.277	0.266	Constant	11.951		4.131	0.001
							Work engagement	0.199	0.527	5.032	0.001

## Discussion

There are many factors in the work environment of a paramedic that may pose significant psychosocial risks. These include: significant emotional burden due to the involvement of paramedics in life-threatening situations and exposure to serious health problems of patients, aggression and violence from patients, their family members and third parties, sleep and eating disorders or musculoskeletal strain resulting from the nature of the work. Most of these threats (22, 23) became significantly visible during the COVID-19 pandemic (1, 24). Taking into account the importance of the issue and the relatively small number of studies in relation to the work of paramedics, own research was undertaken to assess the relationship between job satisfaction and identification with the group of paramedics. The research showed an average overall level of job satisfaction among the surveyed paramedics, and these results are consistent with those obtained in studies conducted in Germany and Korea (25). In the studied group of paramedics the highest level of job satisfaction was demonstrated by respondents aged 35–45, and the lowest by young people aged 25–34. Similar results were obtained in studies conducted by Blau (26). In turn, in the study conducted by Lee et al. (25), the highest satisfaction was reported by respondents aged 30–40, and the lowest by younger people. These results support the findings of other researchers, which demonstrate that with age, employees increasingly value autonomy at work, significantly enhancing their sense of satisfaction. This relationship between autonomy (as a job characteristic) and satisfaction is stronger among employees with more years of age and work experience (27).

Moreover, it was shown that men had higher job satisfaction scores compared to women. The Nawrouzi-Kia study also showed that women reported higher job satisfaction (28), which was not confirmed by own research, which did not show the impact of gender on the level of job satisfaction. The research results taking into account gender are not consistent both in terms of job satisfaction and burnout (28). The study conducted by Basabr et al. showed a negative and significant relationship between gender and job satisfaction, but the comparison concerned men in pre-hospital emergency services and women in hospital settings (29). A study conducted by van der Ploeg et al. (30) showed that factors such as lack of social support from colleagues and superiors, as well as poor communication, turned out to be significant predictors of low job satisfaction, post-traumatic stress disorder, symptoms of burnout and fatigue (31). Many studies have found that support from colleagues is associated with a lower incidence of burnout and a higher level of job and life satisfaction among health care workers (32, 33). Another study showed that low job satisfaction in paramedics is significantly associated with safety outcomes and burnout (33). Aiken et al. found that in hospitals with a positive work environment, nurses showed greater job satisfaction, which translated into better quality of activities performed and safety of care, as well as more satisfied patients. Improving hospital working conditions could be an organizational strategy to improve patient outcomes and retain skilled staff (34). According to Springer (35), employees who feel satisfied in their private lives will tend to be more satisfied with their work, while people who were dissatisfied with their private lives will tended to have a negative assessment of job satisfaction (36). In a study conducted in the United States, 59.4% of surveyed nurses showed high job satisfaction and their satisfaction was positively related to work engagement. Nurses with high job satisfaction were less likely to report the desire to leave the job (37). Duignan, Martin et al. obtained a similar result, also showing the relationship between job satisfaction and commitment, paying particular attention to the need to create an appropriate work environment in which nurses would be supported by their colleagues and superiors (38). Lack of job satisfaction and difficult working conditions accounted for a high rate of attrition among paramedics in Israel (4). Research conducted in Poland by Rasmus et al. showed that a large number of shifts had a negative impact on the paramedics' health, especially in the case of younger employees, and dissatisfaction with the current financial situation significantly contributes to job dissatisfaction (39). The analysis of own research showed that job satisfaction positively correlated with identification with the professional group in terms of ingroup ties, cognitive centrality and ingroup affect. This means that the more an employee identified with the group, the higher his or her job satisfaction score assessed via SSP was, and the higher the job satisfaction he or she achieved as measured via MSQ, the more he or she identified with his or her own group. Many studies have confirmed that one's own professional group is an important factor in shaping professional identity and identification with a group (17, 38). According to Johnston et al. (11), a strong professional identity is a known predictor of personal and professional satisfaction and is seen as the basis of successful practice in health professions (40). In turn, a study conducted in one of the hospitals in Libya showed that only 2.8% of medical workers were satisfied with their work, and as many as 36.31% were dissatisfied. The authors of the study identified four factors that had a direct negative impact on job satisfaction, i.e., comfort at work, treatment at work, remuneration and motivation (40). In own study, paramedics' satisfaction was at an average level. Similar results were obtained in the study by Yu et al. (41), showed that respondents with higher education had lower job satisfaction, while those with more than 12 years of work experience had higher job satisfaction (41). According to Li et al., this was probably due to employees with higher education having greater theoretical knowledge, which increased the expectations (42). In own research, the general work engagement of paramedics was high, with greater involvement shown by respondents aged 46–63 when compared to the group aged 35–45. No differences were found between people of different genders in terms of work engagement in the study sample. In Portugal, researchers compared the work engagement of paramedics, nurses and firefighters, where the paramedics constituted the largest group (*n* = 3,887). In the surveyed sample, general work engagement was rated highest by firefighters, including the vigor subscale, which as probably due to the organization of the rescue and firefighting system in Portugal, in which the vast majority of firefighters were unpaid volunteers very closely involved in performing their tasks and may result from readiness to make sacrifices and take risks in order to save life or property (43). In the work of another researcher conducted on a group of 120 nurses working in fifteen hospital wards of the Provincial Hospital in Jelenia Góra, statistical analysis did not show any relationship between age, education level, work experience and work engagement of the surveyed nurses. The impact of management processes on the level of nurses' work engagement was analyzed. The salary was the main factor lowering commitment. Nurses very often indicated work ergonomics as a factor that influenced work engagement, management's interest in nurses' problems and needs, and employment stability. 30% of respondents indicated the involvement of the management staff had an impact on the creation of a good atmosphere at work (44). In turn, in the research conducted by Załuski and Makara-Studzińska, the aim was to learn about the mutual relationships between emotional work, the level of work engagement and the occurrence of burnout syndrome in a group of nurses working in hospital wards. These studies showed that frequent suppression and hiding of negative emotions correlated with limited interpersonal contacts in the workplace, which was negatively perceived by the employee, and was closely related to the expectations toward the workplace and the emotional competences of employees. Frequently occurring suppressed negative emotions lead to a gradual decline in work engagement (45). Statistical analysis in own research showed that social identification positively correlated with the subscales of work engagement. The results obtained may indicate the subjective perception of the possibility of obtaining various forms of help from colleagues. Ellemers et al. conducted an experiment in which they induced subjects to weakly or strongly identify with a group of people invented for the purpose of the study who “reasoned” inductively. Researched people who felt strongly identified with the group were much less willing to change the group that had a higher status, as opposed to people with lower identification (46). Van Vugt and Hart assessed the impact of group identification on loyalty to the own group. The subjects were offered the opportunity to earn much larger amount of money, but the condition was to leave the group created for the experiment. All three studies conducted by researchers showed that people with high social identification decided to leave the group less often (47).

The area related to job satisfaction among medical staff should be of great importance to management. A high level of job satisfaction directly impacts the wellbeing of employees, which is particularly crucial in the demanding medical environment. When medical staff feel good in their workplace, their levels of motivation and engagement increase, leading to higher productivity and efficiency. Satisfied employees are more likely to take initiative, perform their duties better, and maintain positive relationships with patients, which ultimately can lead to better health outcomes. Therefore, investing in initiatives that improve job satisfaction is essential for achieving organizational success in the healthcare sector.

### Research limitations

The limitations of this research are the sample size and method of selecting the group for research covering one region and the duration of the research. The research was conducted during the COVID-19 pandemic, which significantly limited access to the study sample and might have affected the results obtained. The research was cross-sectional. In the future, it is worth undertaking longitudinal research in the group of paramedics.

The strength of this study is the professional group selected for analysis, which has not been the subject of many studies so far, especially in relation to the issues described. Most of the research so far has been conducted among doctors and nurses.

## Conclusions

The studied group of paramedics was characterized by moderate job satisfaction (SSP scores) and work engagement, with a simultaneous high level of job satisfaction (MSQ scores) and social identification with the professional group. Social identification of paramedics positively correlated with job satisfaction and work engagement, which indicates the importance of this factor for the assessment of professional satisfaction. Sociodemographic variables did not significantly differentiate the level of job satisfaction (measured by SSP or MSQ) or work engagement among paramedics. Social identification of paramedics varied depending on gender. Women showed higher levels of cognitive centrality, suggesting the cognitive prominence of the professional group of paramedics in women. Systematic research on job satisfaction of paramedics can provide important information on factors affecting the quality of work performed, which will enable the adaptation of working conditions in order to increase professional satisfaction and improve the standards of medical services provided.

## Data availability statement

The original contributions presented in the study are included in the article/supplementary material, further inquiries can be directed to the corresponding author/s.

## Ethics statement

The studies involving humans were approved by the Bioethics Committee of the Jagiellonian University (No 1072.6120.79.2018). The studies were conducted in accordance with the local legislation and institutional requirements. The participants provided their written informed consent to participate in this study.

## Author contributions

PK: Conceptualization, Data curation, Formal analysis, Investigation, Methodology, Project administration, Writing – original draft. MK: Supervision, Writing – original draft, Writing – review & editing. PS: Data curation, Formal analysis, Methodology, Writing – review & editing. TI: Supervision, Writing – original draft, Writing – review & editing. MA: Conceptualization, Writing – original draft. IM-L: Data curation, Formal analysis, Methodology, Supervision, Writing – original draft, Writing – review & editing.
